# Echocardiographic Assessment of the Aorta: Tips and Pitfalls

**DOI:** 10.1055/s-0040-1721750

**Published:** 2021-03-24

**Authors:** Sarah C. Hull

**Affiliations:** 1 Section of Cardiovascular Medicine, Yale University School of Medicine, New Haven, Connecticut

**Keywords:** echocardiography, aorta, aortic imaging, aortic dissection, aortic root, ascending aorta, transthoracic echocardiography, aortic measurement, guidelines, bicuspid aortic valve

## Abstract

This Yale Aortic Institute lecture provides “tips and pitfalls” regarding echocardiographic assessment of the aorta.

This lecture ( Video 1 ) guides the listener through important details in acquiring and interpreting ultrasound images of the aorta. The points emphasized are the following:

- By convention, the ascending aorta is measured by echo from leading edge to leading edge. “Leading edge” connotes the edge of the aortic wall that is the closest to the probe (at the top of the inverted “V” of the ultrasound image).
- By transthoracic echo (TTE), the leading edges are the outer anterior wall and inner posterior wall. By transesophageal echo (TEE), the leading edges are the outer posterior wall and inner anterior wall.
- Aortic measurements should be taken (by convention) in diastole (when the aorta is moving least).
- Simple TTE is 70 to 85% sensitive in diagnosing ascending aortic dissection. TEE sensitivity approaches 100%, though the tracheal carina imposes a blind spot on TEE, impeding visualization of distal ascending aorta and proximal aortic arch.
- While computed tomography angiography may be superior for defining full anatomic extent of aortic dissection, echocardiography is superior in assessing functional consequences such as mechanism and severity of aortic regurgitation, evidence of myocardial ischemia when complicated by coronary dissection, or evidence of tamponade physiology when pericardial effusion is present.
- Reverberation artifact can mimic a dissection flap. A true flap moves independently of the outer aortic wall which can be confirmed by one-dimensional motion mode. Color flow respects a true flap but does not respect a reverberation artifact.
- Assessment can be done for bicuspid aortic valve morphology in systole, not diastole. In diastole, when the valve is closed, the raphé can make a bicuspid valve appear as trileaflet. Doming in the parasternal long axis (PLAX) view and an eccentric closure line on PLAX M-mode should also raise suspicion for bicuspid aortic valve.

**Video 1** The Yale Aortic Institute lecture by Dr. Sarah Hull on the “tips and pitfalls” of echocardiographic assessment of the aorta.


